# BRD7 enhances the radiosensitivity of nasopharyngeal carcinoma cells by negatively regulating USP5/METTL3 axis-mediated homologous recombination repair

**DOI:** 10.7150/ijbs.100833

**Published:** 2024-11-11

**Authors:** Mengna Li, Jianxia Wei, Changning Xue, Shipeng Chen, Xiangting Zhou, Lemei Zheng, Yumei Duan, Hongyu Deng, Songqing Fan, Wei Xiong, Faqing Tang, Ming Zhou

**Affiliations:** 1 NHC Key Laboratory of Carcinogenesis, Hunan Key Laboratory of Oncotarget Gene, Hunan Cancer Hospital/the Affiliated Cancer Hospital of Xiangya School of Medicine, Central South University, Changsha 410078, China.; 2 Cancer Research Institute and School of Basic Medical Sciences, Central South University, Changsha 410078, China.; 3 The Key Laboratory of Carcinogenesis and Cancer Invasion of the Chinese Ministry of Education, Central South University, Changsha 410078, China.; 4 Department of Clinical Laboratory, Hunan Cancer Hospital/the Affiliated Cancer Hospital of Xiangya School of Medicine, Central South University, Changsha 410031, China.; 5 Department of Pathology, the Second Xiangya Hospital, Central South University, Changsha 410011, China.

**Keywords:** Nasopharyngeal carcinoma, BRD7, METTL3, USP5, Radiosensitivity, Ubiquitin-proteasome.

## Abstract

An important reason for the poor prognosis of nasopharyngeal carcinoma (NPC) patients is radioresistance. Our previous studies demonstrated that BRD7 is expressed at low levels in NPC and functions as a tumor suppressor to inhibit NPC progression and metastasis. However, the role and mechanism of BRD7 in the development of radioresistance in NPC cells remain unclear. In this study, we first found that BRD7 was lowly expressed in radioresistant NPC tissues and cells compared to radiosensitive tissues and cells and that overexpression of BRD7 promoted the induction of DNA double-strand breaks and increased radiosensitivity in NPC cells. Mechanistically, BRD7 competitively inhibits the binding of the deubiquitinating enzyme USP5 to METTL3, thereby reducing the protein stability of METTL3 through the ubiquitin-proteasome pathway. Furthermore, METTL3 was confirmed to suppress the induction of DSBs and promote the development of NPC radioresistance by regulating BRCA1- and RAD51-mediated homologous recombination repair. Moreover, high BRD7 expression and low METTL3 expression are positively correlated with radiosensitivity and good prognosis in NPC patients. Taken together, our findings reveal that BRD7 promotes the radiosensitization of NPC cells by negatively regulating USP5/METTL3 axis activity and indicate that targeting the BRD7/METTL3 axis might be a novel therapeutic strategy for NPC radiosensitization.

## Introduction

Nasopharyngeal carcinoma (NPC) is one of the most common malignant neck and head tumors in southern China and Southeast Asia 1, 2. The cure rate exceeding 90% for NPC is possible with early radiotherapy 3. Regrettably, the occult nature of early NPC symptoms often leads to diagnosis at advanced stages or after metastasis, thereby impeding timely intervention and resulting in delayed detection in the majority of patients 4. Therefore, comprehensive investigations into the underlying mechanisms of radioresistance are urgently needed, as is the exploration of efficacious therapeutic strategies and targets for NPC 5, 6.

The bromodomain-containing protein 7 (*brd7*) gene, which was cloned in our laboratory using complementary DNA (cDNA) representational difference analysis, has been identified as a tumor suppressor gene in multiple cancers, especially NPC 7. Our previous results confirmed that BRD7 is widely involved in malignant biological processes in NPC, such as cell proliferation, cell cycle progression, apoptosis, invasion and metastasis, by negatively regulating the Ras/MEK/ERK, Rb/E2F, Wnt/β-catenin and PTEN/AKT signaling pathways 8, 9. In addition, BRD7 negatively regulates the transcriptional activity and expression of BIRC2 by targeting the activation of the BIRC2 enhancer region 10. However, the specific role of BRD7 in conferring resistance to radiotherapy has not been determined. Ionizing radiation (IR) induces apoptosis in proliferating cancer cells by inducing the formation of replicative DNA double strand breaks (DSBs) 11, the main type of DNA damage. Kaishun et al. reported that BRD7 participates in the DNA damage response (DDR) and is recruited to damaged chromatin via ATM signaling 12. These findings suggested that BRD7 is closely related to radiotherapy-induced DNA damage in NPC cells.

METTL3 is an essential methyltransferase involved in N6-methyladenosine (m6A) modification and participates in almost all cellular activities 13, including tumor proliferation 14, angiogenesis 15, metastasis 16 and immunity 17. Previous studies have shown that METTL3 is a crucial protein involved in DNA damage repair 18. The phosphokinase ATM can phosphorylate the S43 residue of METTL3 and localize METTL3 to DSBs, leading to m6A modification and protection of nascent RNA, then m6A-modified RNAs form DNA-RNA hybrids at DSBs, thereby modulating HR-mediated DSBR through enhanced recruitment of repair-related proteins such as BRCA1 and RAD51^19^, ^20^, these findings elucidate the role of m6A modification in HR-mediated DSB repair. Our previous studies revealed that BRD7 negatively regulates the protein stability of METTL3 in NPC cells, an effect most likely closely related to BRD7-mediated sensitization to radiotherapy. Therefore, in this study, we conducted an in-depth investigation into the regulatory relationship between BRD7 and METTL3 and revealed the mechanism of BRD7 and METTL3 in DNA damage repair-mediated radioresistance in NPC.

## Methods

### Patients and tumor tissue samples

Tumor samples were obtained from patients with pathologically confirmed NPC (n = 124) at the Second Xiangya Hospital of Central South University. Radiosensitive patients were defined as those without local residual lesions > 3 months and without local recurrent disease > 12 months after completion of radiotherapy. On the basis of the above criteria, the 121 NPC patients were divided into a group of 34 radioresistant patients and a group of 87 radiosensitive patients. The study procedure was approved by the Ethics Committee of Central South University and complied with the Declaration of Helsinki.

### Cell culture

Human NPC cell lines (CNE2, 5-8F) were obtained from the Cell Center of Central South University (Changsha, China) and cultured in RPMI-1640 medium (BI, Kibbutz Beit Haemek, Israel)) supplemented with 10% fetal bovine serum (FBS, BI). The NPC radioresistant cell line CNE2-IRR was provided as a gift from the Molecular Medicine Research Center of Xiangya Hospital 21. HEK293T cells purchased from the American Type Tissue Culture Collection (ATCC) were cultured in DMEM (BI) supplemented with 10% FBS. All cell lines were cultured at 37 °C in a humidified atmosphere with 5% CO_2_.

Stable overexpression of METTL3 and BRD7 5-8F and CNE2 cell lines were constructed as previously described 10. Briefly, the pCDH-copGFP/METTL3 or pCDH-copGFP/BRD7 vector, along with the envelope plasmid pMD2.G and the packaging plasmid psPAX2 (at a ratio of 4:1:3), were co-transfected into HEK293FT packaging cells in serum-free medium using Lipofectamine 3000 reagent (Invitrogen, Carlsbad, USA) according to the manufacturer's protocol. After 48 hours, virus particles were collected, filtered, and used to transduce the target CNE2 and 5-8F cells. Finally, positive cells were selected via FACS, resulting in cells with stable overexpression of METTL3 and BRD7.

### Clonogenic survival assay

Single-cell suspensions were seeded into 6-well plates at a density of 2000 cells per well. The cells were treated with X-ray radiation at doses of 0, 2, 4, and 8 Gy until they adhered to the plates, after which they were cultured for 10-14 days. Subsequently, the cell colonies were stained with crystal violet and counted. The survival rate in each group was calculated according to the corresponding plating efficiency, and colonies containing more than 50 cells were counted.

### Western blot analysis

A standard western blotting procedure was used, which involved lysing cells in RIPA cell lysis buffer (NCM Biotech, Suzhou, China) supplemented with 1% protease/phosphatase inhibitors (NCMs) and determination of protein concentrations using a Pierce^TM^ BCA Protein Assay Kit (Thermo Fisher, USA). Samples containing equal amounts of protein were loaded into 10% SDS-PAGE gels, and the separated proteins were then transferred onto 0.2 µM or 0.45 µM PVDF membranes (Merck Millipore, Billerica, MA, USA). The membranes were incubated with specific primary antibodies overnight after blocking with a solution containing 5% skim milk powder for 1 h. The following primary antibodies were used: anti-BRD7 (1:2,000) and anti-GAPDH (1:10,000), purchased from Proteintech Group Inc. (Wuhan, China); anti-HA mouse (1:1,000), anti-γ-H2AX (1:1,000) and anti-USP5 (1:1,000), purchased from ABclonal Technology Co., Ltd. (Wuhan, China); anti-Flag (1:500), purchased from Sigma-Aldrich (USA); anti-METTL3 (1:2,000), purchased from Abways Technology Co., Ltd. (Beijing, China); and anti-Ubiquitin (1:2,000), purchased from Santa Cruz Biotechnology Co., Ltd. (Shanghai, China). The next day, the membranes were incubated with the corresponding HRP-conjugated secondary antibodies (goat anti-rabbit IgG and goat anti-mouse IgG purchased from Proteintech Group Inc.). The proteins were detected using enhanced chemiluminescence (ECL) reagents (Vazyme Biotechnology, Nanjing, China), and immunoreactions were visualized and imaged with a SmartChemi TM system (SAGE, China). Band intensities were quantified via ImageJ software.

### Confocal immunofluorescence microscopy

For assessment of the colocalization of BRD7 and METTL3 by double-label immunofluorescence staining, experiments were conducted as previously described 22. The antibodies used in this study included an anti-BRD7 antibody (1:400; Proteintech) in tandem with an Alexa Fluor 488 secondary antibody (1:1,000; Invitrogen) and an anti-Flag antibody (1:1,000; Sigma‒Aldrich) in tandem with an Alexa Fluor 594 secondary antibody (1:1,000; Invitrogen). Imaging was conducted using a Laica confocal microscope. Fluorescence intensity curves were calculated by Image J.

Immunofluorescence staining to assess foci formation was conducted as follows. Cells were seeded on round coverslips in 12-well plates and treated with IR. Then, the cells were fixed in 4% paraformaldehyde for 15 min, permeabilized in 0.3% Triton X-100 for 10 min, blocked in 5% goat serum at room temperature for 30 min and incubated with the following primary antibodies at 4 °C overnight: anti-γ-H2AX (Ser139) (1:500, CST), anti-METTL3 (1:400, Abways), anti-Rad 51 and anti-BRCA1 (1:200, ABclonal). The cells were washed with PBS and incubated with the corresponding secondary antibodies for 1 h in the dark. Subsequently, DAPI staining was performed to visualize nuclear DNA. Then, we used a confocal scanning microscope (Leica, Barbarano Vicentino VI, Italy) to acquire images.

### Plasmid construction and transfection

The Flag-tagged METTL3 (Addgene, #53739) plasmid was purchased from Addgene (https://www.addgene.org/). The HA-tagged USP5 and BRD7 plasmid were cloned and inserted into the pCMV-HA vector to construct the corresponding overexpression plasmids. Plasmids containing fragments of BRD7 (pCMV-HA-N-Ter, HA-C-Ter, HA-ΔN, HA-ΔBRD, and HA-ΔC) were constructed by our laboratory, and plasmids containing fragments of METTL3 (pCDNA3.1-Flag-ΔmTase, Flag-mTase) were subcloned and inserted into the pCDNA3.1-Flag vector. The following procedure was used for construction of the truncation plasmids: the target gene fragments were amplified via PCR, and the pCDNA3.1-Flag vectors were digested with the XhoI/EcoRI restriction enzymes, and finally, the PCR amplification products were ligated into the digested vectors. The plasmids were verified by DNA sequencing. The structures of the BRD7 and METTL3 truncation mutants are shown in Figure 3 and Figure S2.

The siRNAs used in this study include Control (non-targeting) siRNA, METTL3 siRNA #1 (sequence: 5'-ACUUCUUCUCUAAUUCAGGGU-3´), METTL3 siRNA #2 (sequence: 5'-CUGCAAGUAUGUUCACUAUGA-3').

For transient transfection, the plasmids were transfected into the indicated cells with Polyplus transfection reagent (Illkirch-Graffenstaden, France) according to the manufacturer's protocols.

### *In vivo* ubiquitination assay

Cells were treated with the proteasome inhibitor MG132 (Selleck, Houston, United States) for 4 h. The cells were lysed with immunoprecipitation (IP) buffer sonicated, and the lysates were then centrifuged. The supernatants were subjected to IP with an anti-METTL3 antibody (Abways) at 4 °C with shaking. Subsequently, the precipitates were eluted in SDS sample buffer and subjected to western blot analysis with an anti-Ubiquitin antibody (Santa Cruz).

### RNA m6A dot blot assay

Total RNA was extracted from 5-8F and CNE2 cells and spotted onto nitrocellulose membranes. The membranes were then subjected to crosslinking with ultraviolet light and blocked with 5% milk for 1 h. The membranes were incubated with an anti-m6A antibody (1:1,000; Abcam, Boston, MA, USA) overnight at 4 °C, washed three times with triethanolamine-buffered saline-Tween (TBST), and subsequently incubated with goat anti-rabbit IgG-HRP as a secondary antibody at room temperature for 1 h. The membranes were then washed with TBST and visualized using an imaging system. Another membrane was spotted and stained with 0.02% methylene blue (Sigma-Aldrich) to ensure baseline consistency among the groups.

### Apoptosis assay

Apoptosis was detected via FACS analysis with Annexin V-FITC/PI double staining. Briefly, cells were collected after treatment with or without IR, allowed to recover at 37 °C for 24 h for detection of apoptosis, and then stained with the components of an Annexin V-FITC/PI apoptosis kit (BD Biosciences, NJ, USA). The data were analyzed using FlowJo v10 (FlowJo, LLC, USA).

### RNA extraction and RT-qPCR

Total RNA was isolated with TRIzol reagent (Invitrogen, Carlsbad, USA), complementary DNA was reverse transcribed with a RevertAid First Strand cDNA Synthesis Kit (K1622; Thermo Scientific, Waltham, USA) according to the manufacturer's protocols, and qPCR was performed with SYBR Green reagent (Accurate Biotechnology, Hunan, China) and a Bio-Rad CFX96 Touch sequence detection system (Bio-Rad Laboratories, Inc.). Target gene expression is expressed as a ratio to housekeeping gene GAPDH expression. The following primers 5' to 3' were used: BRD7 Forward: AGAATAAGAAGTGGGAGCAGAAGC, BRD7 Reverse: ACGAGGTGAGTTCTCTCCAATGAT; METTL3 Forward: CCTTAACATTGCCCACTGATG, METTL3 Reverse: GCTTTCTACCCCATCTTGAGTG; GAPDH Forward: CGAGATCCCTCCAAAATCAA, GAPDH Revers: TTCACACCCATGACGAACAT. The relative fold changes in expression were calculated using the 2^-ΔΔCT^ method, and each sample was analyzed in triplicate.

### Comet assay

Cells were treated with IR (6 Gy) and harvested 24 hours after IR. We used a comet assay kit (KeyGen Biotech, Nanjing, China) according to the manufacturer's instructions. Briefly, cells were exposed to 6 Gy irradiation and subjected to the comet analysis at specified time points. After staining with propidium iodide (PI), comets were imaged using fluorescence microscopy, 10 comet tails were analyzed for each group, and ImageJ software was used to analyze the tail moments.

### DNA repair assay

Integrated DNA repair reporter systems were used to determine the efficiency of homologous recombination 23. Briefly, cells were transiently transfected with the indicated plasmids and then cotransfected with direct repeat GFP (DR-GFP) or EJ5-GFP reporters. Subsequently, the cells were transfected with the I-Sce-I expression vector pCBASceI (Addgene). Forty-eight hours after transfection, the percentage of GFP-positive cells was determined via flow cytometry. HR and NHEJ efficiencies are presented as percentages relative to control cells. The frequencies shown in the figures are presented as the mean ± SD of three independent experiments.

### Animals

BALB/c nude mice (4-5 weeks old; female; 15-18 g) were purchased from Hunan Slake Jingda Experimental Animal Co., Ltd. For the xenograft models, we divided the 20 BALB/c nude mice into four groups: 5-8F/Ctrl, 5-8F/BRD7, 5-8F/METTL3 and 5-8F/BRD7 plus METTL3 (five mice in each group). A total of 5×10^6^ cells in 0.9% saline solution with Matrigel (total volume of 150 µL) were injected into the axilla of each mouse, and the mice were checked every 2 days. Once the tumors became palpable (100-150 mm^3^), the mice were treated with 6 Gy IR. The tumor volume was calculated as tumor volume = (length×width^2^)/2. Tumor growth curves were plotted. All mice in the four groups were sacrificed 18 days after subcutaneous inoculation, and all tumors were excised, weighed, fixed with 4% formaldehyde and then embedded in paraffin for IHC staining.

### Immunohistochemistry and scoring

Primary tumor sections embedded in paraffin were deparaffinized and rehydrated using xylene and alcohol gradients. Antigen retrieval was carried out using an improved citrate antigen retrieval solution (pH = 6.0) prior to incubation with 3% H_2_O_2_ for 15 min to inhibit endogenous peroxidase activity. The sections were then incubated overnight at 4 °C with anti-BRD7, anti-METTL3, anti-γ-H2AX antibodies (1:100; CST), after which proteins were detected using an UltraSensitiveTM SP (Mouse/Rabbit) IHC Kit (MXB Biotechnology, KIT-9720) according to the manufacturer's instructions. The intensity of staining was scored on a four-point scale: 0 (no brown particulate staining), 1 (light brown particulate staining), 2 (moderate brown particulate staining) and 3 (dark brown particulate staining). The percentage of positive tumor cells was scored on a four-point scale: 1 (< 25% positive cells), 2 (26-50% positive cells), 3 (51-75% positive cells) and 4 (> 75% positive cells). The two scores were multiplied to obtain the IRS (ranging from 0 to 12), which was used to determine whether expression was high (score ≥ 6) or low (score < 6) 22, 24.

### Statistical analysis

GraphPad Prism 7.0 (CA, USA) was utilized for statistical analysis. Significance was determined using a two-tailed unpaired Student's test. All the data used in this study met the assumptions of the statistical tests. The mean ± SD. values are presented for all the quantitative data as indicated. A statistically significant difference was defined as *P* < 0.05. All the experiments were performed at least three times.

## Results

### BRD7 promotes radiation-induced double-strand breaks and increases radiosensitivity in NPC cells

**Figure 1:**
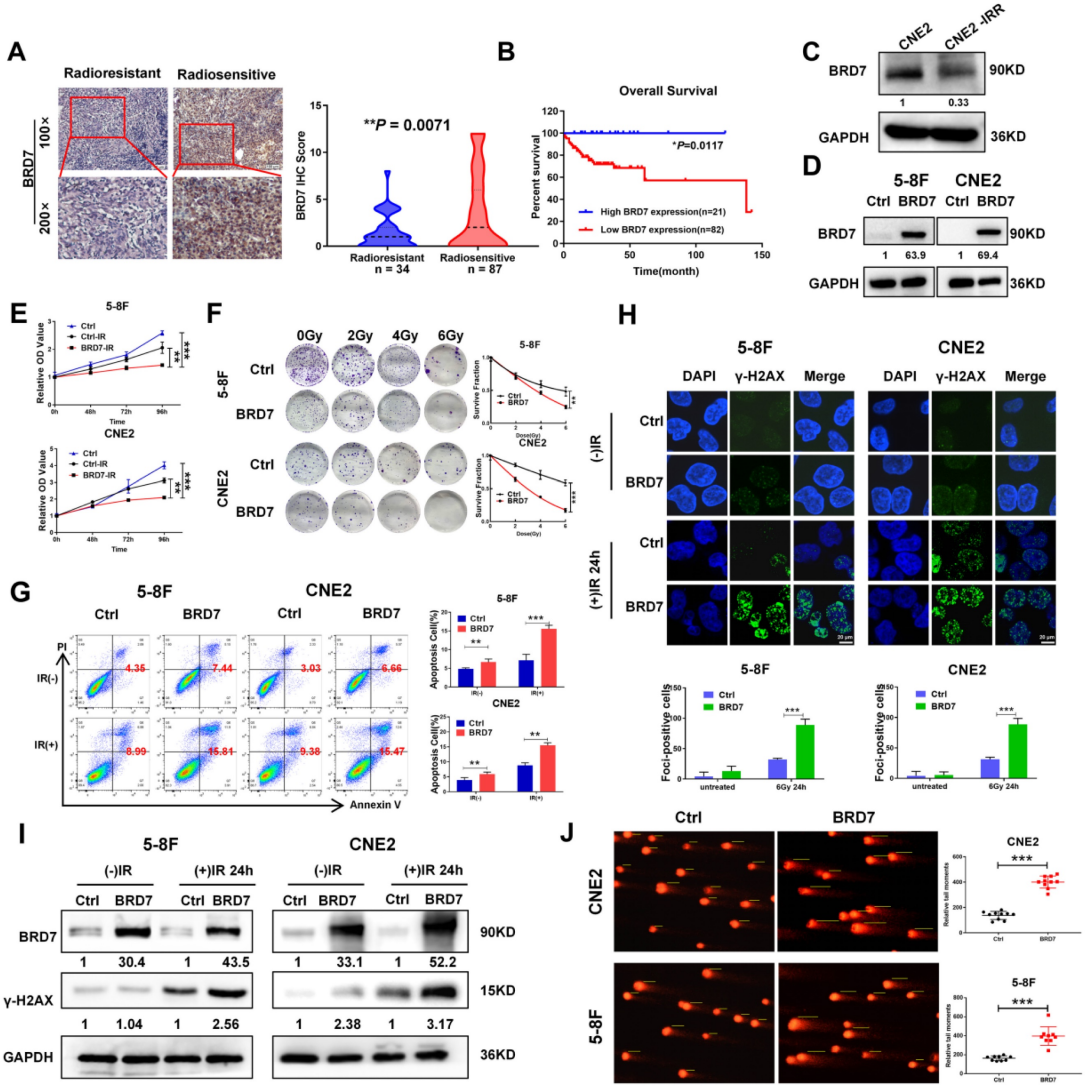
** BRD7 inhibits the radioresistence of NPC cells by increasing DNA damage.** (A) IHC assay shows the levels of BRD7 in the radiosensitive and radioresistant NPC tissues, respectively. Original magnification, 100×; scale bars represent 100 μm. (B) Impact of BRD7 expression on prognosis of patients with NPC. (C) Western blotting shows the protein expression level of BRD7 in radiosensitive and radioresistant cells. (D) Western blotting was performed to detect the BRD7 protein level using antibodies against BRD7, and GAPDH served as an internal control. (E) CCK-8 assay was performed to detect the cell viability of 5-8F and CNE2 cells with stable overexpression of BRD7. (F) Colony formation assay and quantification analysis of NPC cells with BRD7 before and after irradiation were conducted, along with the examination of the effects of BRD7 on the survival rate of NPC cells after radiotherapy. Nonlinear regression (curve fit) was used to determine the survival fraction at different doses based on the formula for Survival Fraction. (G) Flow cytometry analysis of cell apoptosis via Annexin V-FITC/PI double staining showed representative (left) and statistical results (right). (H) The levels of γ-H2AX at different times after 6Gy irradiation were detected by immunofluorescence in 5-8F and CNE2 cells. Cells displaying ten or more foci were counted as positive. Histogram of the percentage of γ-H2AX foci in the four groups of NPC cells. (I) Detection of γ-H2AX protein levels of 5-8F and CNE2 cells treated with or without 6 Gy of IR. (J) Representative comet images of 5-8F and CNE2 cells stable transfection with BRD7 treated with 4Gy and subjected to alkaline single-cell electrophoresis. DNA damage was measured in the comet tail. Error bars represent the mean ± SD. *P < 0.05; **P < 0.01; ***P < 0.001. All experiments were performed in triplicate.

Our previous study demonstrated that BRD7 is downregulated in NPC and exerts a tumor-suppressive effect by inhibiting the malignant progression and metastasis of NPC. In this study, we first showed that BRD7 expression was significantly reduced in tissues from radioresistant NPC patients compared to those from radiosensitive NPC patients (Figure 1A) and that high BRD7 expression was found to be a favorable prognostic factor for patient survival (Figure 1B). Next, we measured the expression of BRD7 in the radiotherapy-resistant NPC cell line CNE2-IRR and its radiosensitive parental cell line CNE2 by western blot analysis. The results showed that BRD7 was significantly downregulated in the radioresistant CNE2-IRR cells (Figure 1C). Collectively, these results showed that downregulation of BRD7 might be the molecular mechanism underlying the resistance of NPC cells to radiotherapy. Next, we established cell lines with stable BRD7 overexpression through lentiviral transduction in the NPC cell lines 5-8F and CNE2 (Figure 1D). Subsequent CCK-8 and clonogenic survival assays revealed that overexpression of BRD7 resulted in a significant decrease in radiation-induced cell survival (Figure 1E-F). Moreover, we evaluated the role of BRD7 in IR-induced apoptosis by flow cytometric analysis. As expected,the Annexin V-FITC/PI-based apoptosis assay demonstrated a significant increase in the apoptosis ratio in cells overexpressing BRD7 (Figure 1G). Taken together, these data indicate that BRD7 enhances ionizing radiation-induced apoptosis in NPC cells and increases their radiosensitivity.

To better understand the mechanisms underlying BRD7-induced radiosensitivity in NPC cells, we measured the formation of γ-H2AX foci, which indicates DNA double-strand breaks, by immunofluorescence staining after induction via IR exposure. Compared with that in control cells, the number of γ-H2AX foci in BRD7-overexpressing cells was markedly increased, indicating an increase in DNA damage in these cells (Figure 1H). In addition, western blot analysis showed that the level of the DNA damage-related protein γ-H2AX was considerably higher in cells with BRD7 overexpression (Figure 1I). To further investigate the effect of BRD7 on radiation-induced DNA lesions, a comet assay was performed to detect DSB based on the tail moment, and the results showed that overexpression of BRD7 dramatically increased the tail moment length in NPC cells after induction via 4 Gy irradiation (Figure 1J). Taken together, our data suggest that BRD7 is involved in radiation-induced DSB repair, which might be the critical mechanism by which BRD7 sensitizes cells to radiotherapy.

### BRD7 is an interacting protein of METTL3 that decreases the stability of METTL3 through the ubiquitin-proteasome pathway

**Figure 2:**
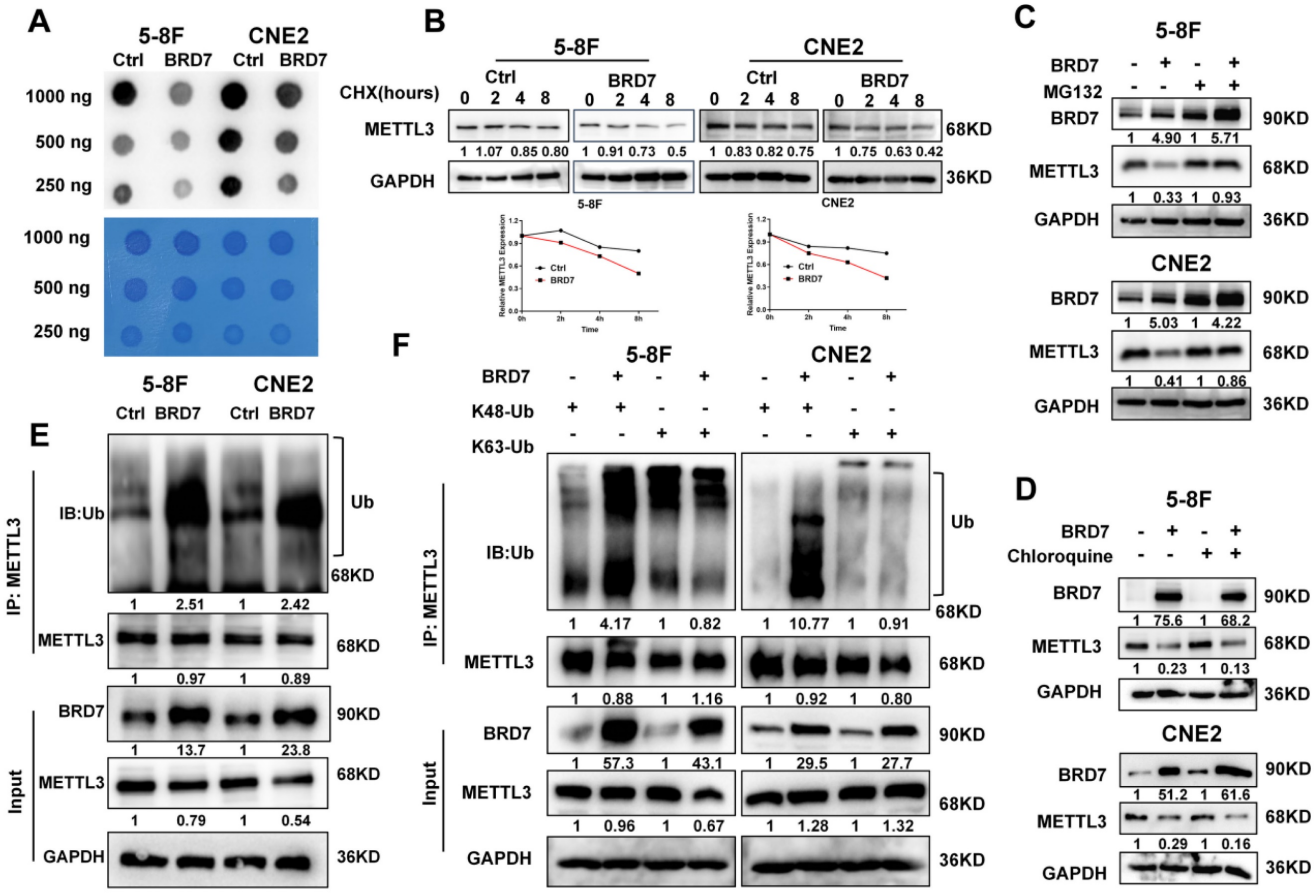
** BRD7 negatively regulates METTL3 stability through the proteasome-dependent pathway.** (A) m6A dot blot analysis of the effects of overexpressing BRD7 on total m6A levels in 5-8F and CNE2 cell lines. (B) BRD7-overexpressed cells were treated with CHX (10 μg/mL) and collected at 0, 2, 4 and 8 h, followed by the calculation of protein decay rate. (C) and (D) BRD7-overexpressed cells were treated with MG132 or CQ (10 μg/mL) 8 h, and the indicated protein levels were examined, GAPDH was used as an internal control. (E) and (F) BRD7 decreased the ubiquitination of METTL3. 5-8F and CNE2 cells overexpressing BRD7 were co-transfected with HA-Ub, HA-Ub-K48 or HA-Ub-K63. Cellular extracts were immunoprecipitated with anti-METTL3 followed by *in vivo* ubiquitination assay analysis of polyubiquitination of METTL3.

An increasing number of studies show that m6A modification plays an important role in the process of DNA damage repair. To further explore the mechanism by which BRD7 promotes IR-induced radiosensitivity and to investigate the relationship between BRD7 and m6A modification in NPC cells, an m6A dot blot assay and mass spectrometry analysis were performed to determine the total RNA m6A modification levels. The results indicated that m6A levels were significantly decreased in BRD7-overexpressing 5-8F and CNE2 cells compared to control cells (Figures 2A and S1A). M6A methylation of RNA is dynamically regulated by methyltransferases and demethylases. To elucidate the regulatory role of BRD7 in modulating m6A modification, we investigated its effect on the expression levels of m6A methyltransferases (METTL3, METTL14, and WTAP) and demethylases (ALKBH5 and FTO) in NPC cells using western blot analysis. The results showed that overexpression of BRD7 markedly suppressed the protein expression of the m6A methyltransferase METTL3, while it had no significant effect on the expression of other m6A regulators (Figure S1B). Additionally, METTL3 mRNA levels did not significantly change with increasing BRD7 expression (Figure S1C), suggesting that BRD7 reduces m6A modification in NPC cells by specifically decreasing the protein level of METTL3. We used the protein synthesis inhibitor cycloheximide (CHX) to examine whether BRD7 decreases METTL3 protein stability. As shown in Figure 2B, METTL3 levels dropped more rapidly with BRD7 overexpression, indicating increased METTL3 degradation. Then, we next determined which pathway is involved in BRD7-mediated degradation of the METTL3 protein. Cells were treated with the lysosomal inhibitor chloroquine (CQ) or the proteasome inhibitor MG132 along with CHX. Western blotting revealed that MG132 but not chloroquine blocked the reduction in METTL3 protein expression resulting from BRD7 overexpression, suggesting that BRD7 may reduce the stability of the METTL3 protein through the ubiquitin-proteasome pathway (Figure 2C-D). Moreover, an *in vitro* ubiquitination assay showed that BRD7 promoted the ubiquitination of METTL3 (Figure 2E). Lysine 48 (K48) and lysine 63 (K63)-linked polyubiquitination polymers are the predominantly ubiquitin chains found on cellular proteins. Therefore, we transfected HA-Ub K48 or HA-Ub K63 into 5-8F and CNE2 cells with BRD7 overexpression. The co-IP assay results showed that the ubiquitination of METTL3 was catalyzed by BRD7 mainly at position K48 but not K63 (Figure 2F). These results suggest that BRD7 decreases the protein stability of METTL3 via the ubiquitin-proteasome pathway.

**Figure 3:**
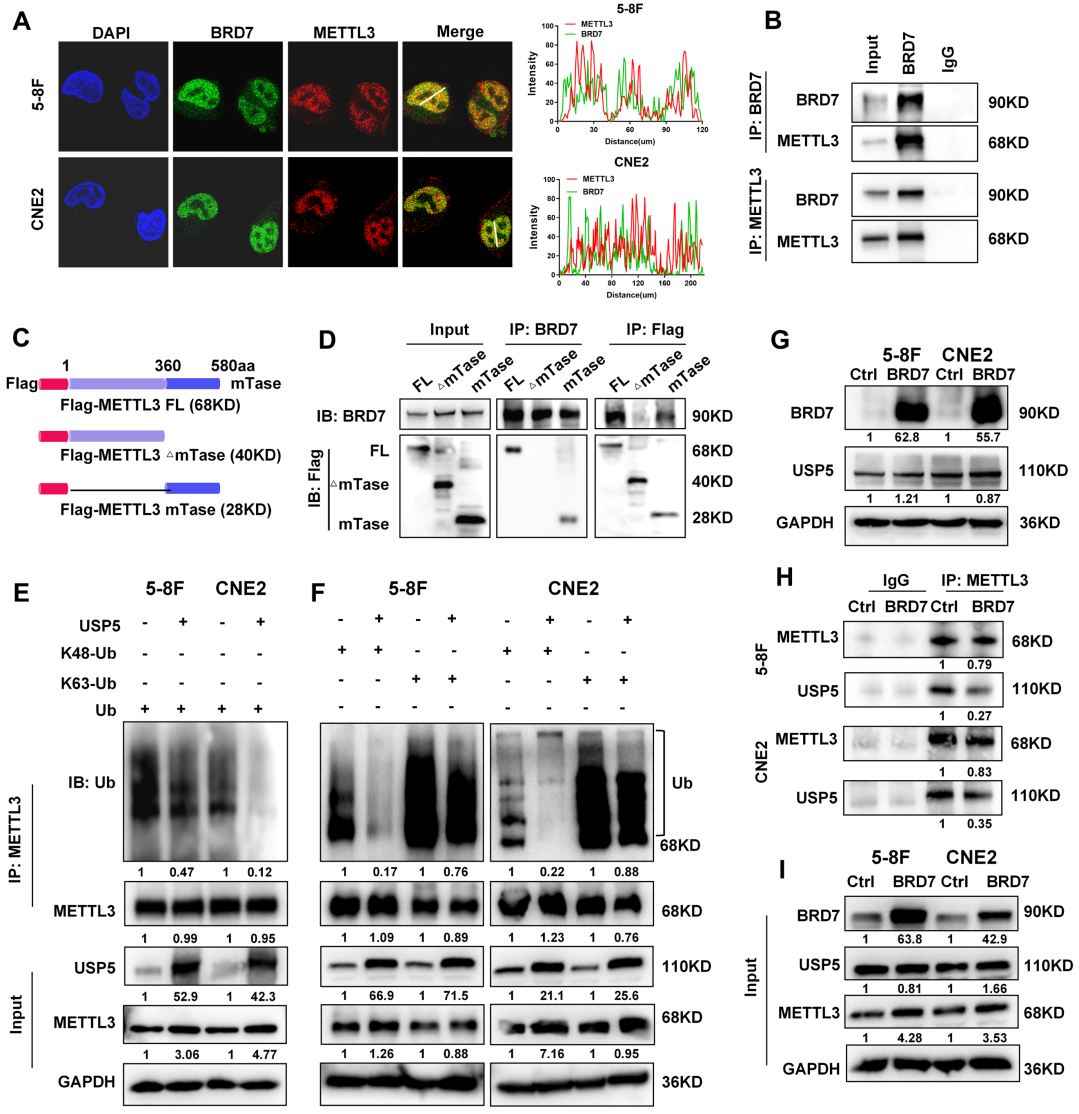
** BRD7 and USP5 competitively bind the methyltransferase domain of METTL3.** (A) Immunofluorescence experiment was used to detected the BRD7 and METTL3 for the subcellular colocalization in 5-8F and CNE2 cells. (B) Co-IP assays and western blotting confirmed the interaction between BRD7 and METTL3 in NPC cells. (C) Structures of methyltransferase domains of METTL3. (D) Co-IP assays and western blotting confirmed the interaction region between BRD7 and METTL3. (E) and (F) Analysis of METTL3 ubiquitin chain types that are catalyzed by USP5 in 5-8F and CNE2 cells expressing USP5 were co-transfected with HA-Ub, HA-Ub-K48 or HA-Ub-K63. (G) Western blotting was performed to detect the BRD7 and USP5 protein level using antibodies against BRD7 and USP5, GAPDH served as an internal control. (H) and (I) Co-IP assays and western blotting confirmed that BRD7 inhibits USP5 to interact with METTL3.

To further investigate the mechanism by which BRD7 regulates METTL3, immunofluorescence and coimmunoprecipitation assays were performed to evaluate the interaction between the BRD7 and METTL3 proteins. The BRD7 and METTL3 proteins were found to colocalize in the nucleus of NPC cells, and BRD7 and METTL3 could be reciprocally immunoprecipitated (Figure 3A, B). Additionally, several domain truncation mutants were constructed based on the results of structural analysis to further investigate the interaction between BRD7 and METTL3. A schematic diagram of the full-length and truncated BRD7/METTL3 constructs is shown in Figures 3C and S2A. The results revealed that the N-terminal domain of BRD7 and the methyltransferase (mTase) domain of METTL3 provided the structural basis for their interaction (Figure 3D, S2B-D). These results demonstrated that METTL3 is an interacting protein of BRD7 and that BRD7 can bind to the mTase domain of the METTL3.

Ubiquitination is facilitated by ubiquitin E3 ligase enzymes and counteracted by deubiquitinases (DUBs). As USP5 was previously identified as a potential deubiquitinase of METTL3 in HCC 25, we further investigated the regulatory association between USP5 and METTL3 in NPC cells. The results showed that overexpression of USP5 increased the protein level and stability of METTL3 in 5-8F and CNE2 NPC cells (Figure S3A-B) and decreased the ubiquitination of METTL3 (Figure 3E), However, overexpression of USP5 dramatically decreased K48-linked ubiquitin chain but not the K63-linked of METTL3 (Figure 3F), suggesting that USP5 deubiquitinates K48-linked ubiquitin chain of METTL3. Moreover, the results of immunofluorescence and co-IP experiments demonstrated that USP5 and METTL3 interact and colocalize with each other in NPC cells (Figure S3C-D), and the co-IP experiments also demonstrated that the mTase domain of METTL3 provides the structural basis for the interaction of METTL3 with the USP5 protein (Figure S3E). These results confirm that USP5 functions as a potential deubiquitinase of METTL3, enhancing its protein stability in NPC cells through its mTase domain. Furthermore, we found that overexpressed BRD7 did not significantly alter the protein expression level of USP5. Instead, it competed with USP5 for binding to the mTase domain of METTL3 (Figure 3G-I), thereby inhibiting METTL3's binding to USP5 and reducing the protein stability of METTL3 in NPC cells.

### BRD7 prohibits METTL3-mediated DNA repair through the homologous recombination repair pathway

**Figure 4:**
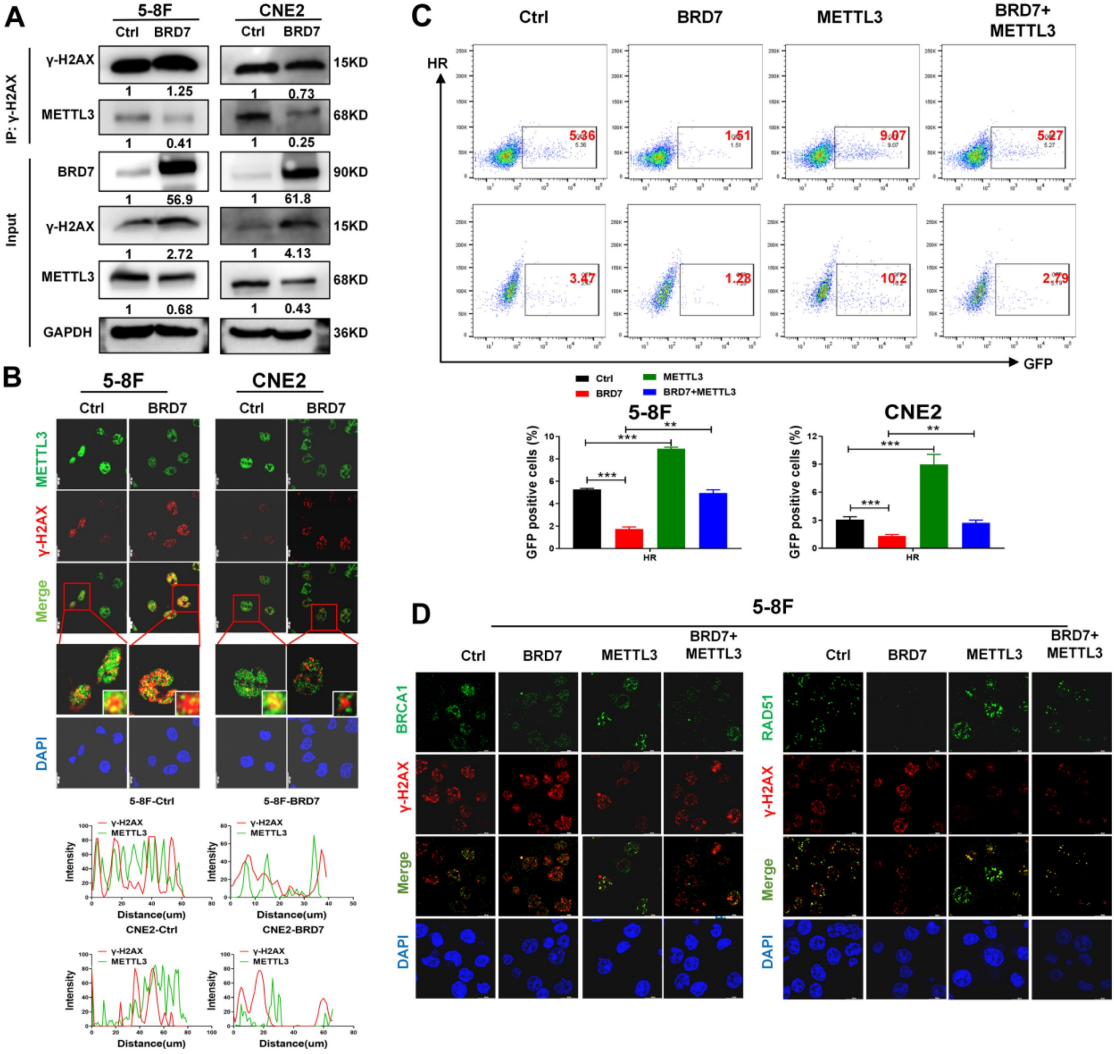
** BRD7 inhibition of METTL3-mediated homologous recombination repair in DSBs** (A) and (B) Co-IP and immunofluorescence experiments detected the METTL3 expression in the DNA damage site (γ-H2AX). (C) Relative HR repair efficiency in 5-8F and CNE2 cells stably expressing the BRD7 and METTL3, respectively. Data are means ± SD from three independent experiments. **P < 0.01; ***P < 0.001. (D) Immunofluorescence assay to examine recruitment of repair factors BRCA1 or RAD51 to the DSB site (marked by γ-H2AX).

Evidence is presented that METTL3-m6A-YTHDC1 axis promotes accumulation of DNA-RNA hybrids at DSB sites, which then recruit RAD51 and BRCA1 for HR repair 19. Therefore, immunofluorescence staining and immunoprecipitation assays were performed to investigate the effects of BRD7 and METTL3 on DNA damage and repair. These results also showed that METTL3 interacted with γ-H2AX and localized to sites of IR-induced DNA damage in NPC cells and that overexpression of BRD7 inhibited the accumulation of METTL3 at sites of IR-induced DNA damage (Figure 4A-B), suggesting that the BRD7/METTL3 axis is required for the repair of IR-induced DSBs. To determine which pathway of DSB repair involves BRD7/METTL3, HR and NHEJ assays were conducted using the DR-GFP and EJ5-GFP systems in NPC cells (Figure S4A). Our data showed that overexpression of BRD7 in cells containing the DR-GFP and EJ5-GFP reporters caused a significant reduction in the number of GFP-positive cells compared to the control sample. In contrast, cells overexpressing METTL3 exhibited significantly increased HR efficiency compared to control cells, while caused no significant effect on the NHEJ repair. Furthermore, the restoration of METTL3 expression significantly reversed the inhibitory effect of BRD7 on HR repair, but did not affect NHEJ efficiency (Figure 4C, S4B). These results support that BRD7 promotes radiosensitization of NPC at least partly through METTL3-mediated HR repair. BRCA1 and RAD51 are key HR factors that are recruited to DSBs to promote subsequent HR-mediated DSBR. Previous study reported that knockdown of METTL3 interferes with recruitment of RAD51 and BRCA1 to DSBs 19. Furthermore, in our study, the immunofluorescence assays also showed that BRD7 inhibited the recruitment of the HR repair factors BRCA1 and RAD51 to DSB sites (Figure 4D, S4C). These results suggest that BRD7 prohibits METTL3-mediated HR repair by inhibiting the recruitment of METTL3 to the key repair factors BRCA1 and RAD51.

### METTL3 promotes radioresistance in NPC cells

**Figure 5:**
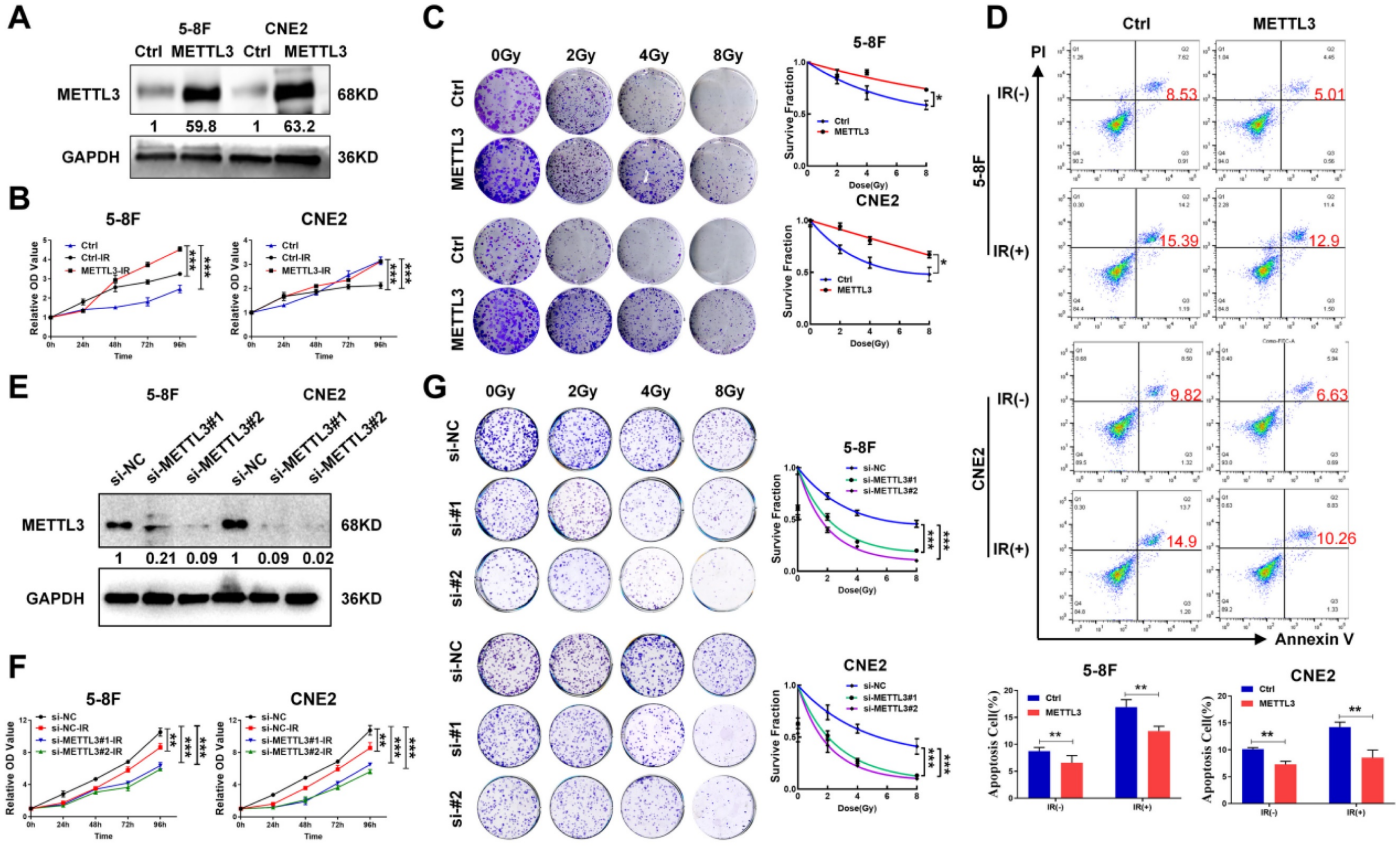
** METTL3 expression is elevated in radioresistant NPC cells and promotes resistance to radiotherapy.** (A) Western blotting showed the protein expression level of METTL3 in 5-8F and CNE2 cells, and GAPDH served as an internal control. (B) CCK-8 assay was performed to detect the cell viability of 5-8F and CNE2 cells with stable overexpression of METTL3 after irradiation. (C) Colony formation and survival curves of 5-8F and CNE2 cells stable transfected with METTL3 were measured after X-ray irradiation at different doses (0, 2, 4, and 8 Gy). The nonlinear regression (curve fit) survival fraction at different doses was curved based on the formula of Survival Fraction. (D) Flow cytometry analysis of cell apoptosis via Annexin V-FITC/PI double staining showed representative and statistical results. (E) Western blotting showed the protein expression level of METTL3 in 5-8F and CNE2 cells, and GAPDH served as an internal control. (F) CCK-8 assay and (G) survival curves of 5-8F and CNE2 cells with METTL3 knockdown were measured after X-ray irradiation at different doses (0, 2, 4, and 8 Gy). The nonlinear regression (curve fit) survival fraction at different doses was curved based on the formula of Survival Fraction. Error bars represent the mean ± SD. *P < 0.05; **P < 0.01; ***P < 0.001. All experiments were performed in triplicate.

BRD7 prohibits METTL3-mediated HR repair by decreasing the recruitment of key repair factors to sites of IR-induced DNA damage, suggesting that the BRD7/METTL3 axis might be the critical mechanism of NPC radioresistance. To confirm this hypothesis, NPC cell lines with stable ectopic expression of METTL3 were constructed. The successful overexpression of METTL3 was validated by western blotting (Figure 5A). Subsequent, the CCK-8 and clonogenic survival assays revealed that overexpressing METTL3 resulted in a significant increase in cell survival after induction by irradiation (Figure 5B-C). In addition, the Annexin V-FITC/PI-based apoptosis assay also showed that the apoptosis rate of NPC cells overexpressing METTL3 was significantly decreased (Figure 5D). Correspondingly, we also used siRNA targeting METTL3 to construct METTL3 knockdown cells (Figure 5E), CCK-8 and clonogenic survival assays revealed that METTL3 knockdown promoted the radiosensitivity of NPC cells after radiotherapy (Figure 5F-G). Collectively, these findings provide substantial evidence supporting the role of METTL3 in increasing radioresistance in NPC cells.

### Restoring METTL3 expression reverses the radiosensitizing effect of BRD7 in NPC cells

**Figure 6:**
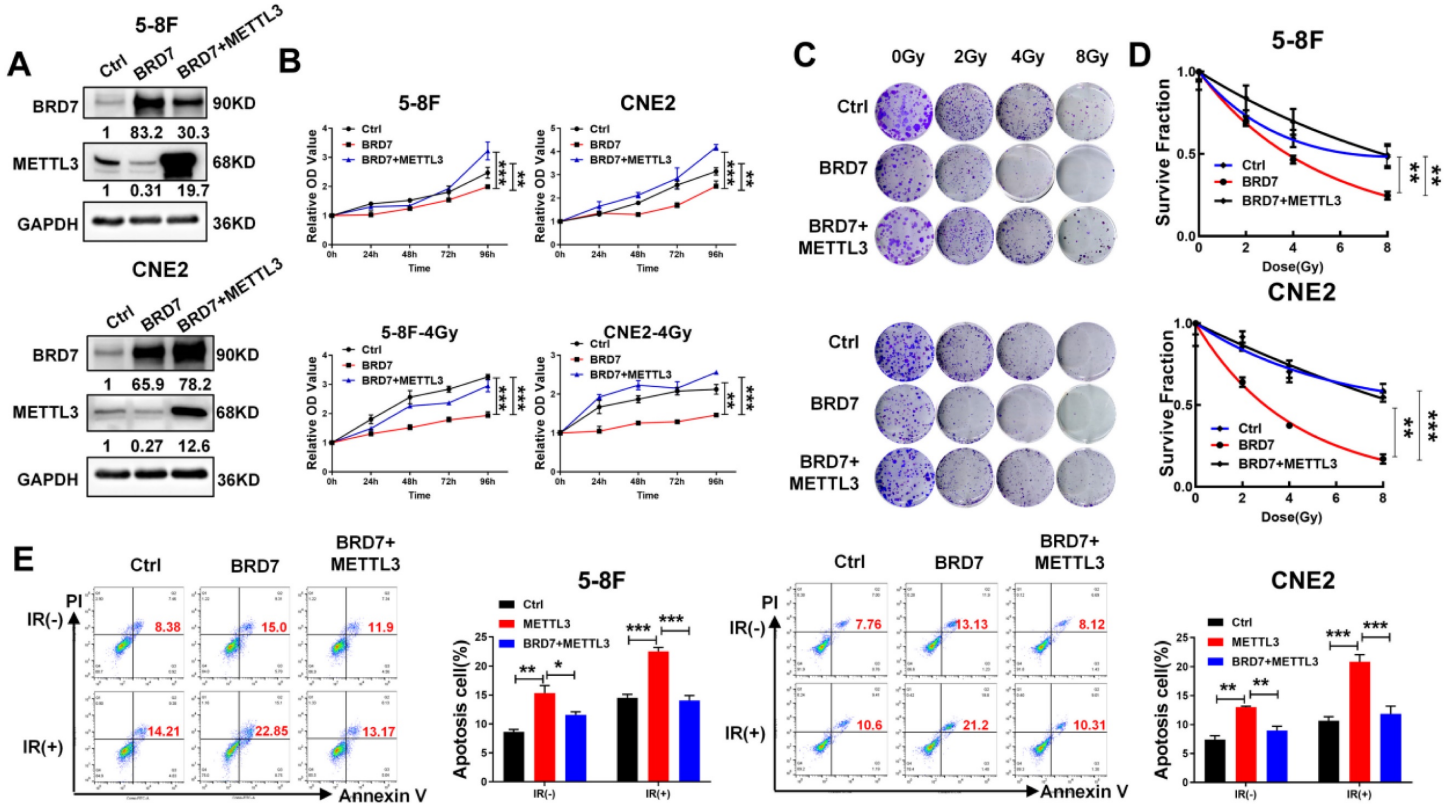
** Restoration of METTL3 expression reverses the radioresistive inhibitory effect of BRD7.** (A) Western blotting was performed to confirm BRD7 and METTL3 protein levels using antibodies against BRD7 and METTL3. GAPDH served as an internal control. (B) CCK-8 analysis to detect proliferation in before and after irradiation 5-8F and CNE2 cells stably with BRD7 overexpression, BRD7, and METTL3 simultaneous overexpression or control group. (C) Colony formation assay to detect the colony formation number of cells after 0, 2, 4, and 8 Gy irradiation in different cells. (D) The nonlinear regression (curve fit) survival fraction at different doses was curved based on the formula of Survival Fraction. (E) Flow cytometry to detect the apoptosis of 5-8F and CNE2 cells after 4 Gy irradiation. Error bars represent the mean ± SD. *P<0.05, **P<0.01, ***P<0.00. All experiments were performed in triplicate.

As METTL3 expression is negatively regulated by BRD7 at the posttranslational level and promotes NPC radioresistance, the focus of this study was shifted towards confirming the essential function of METTL3 in BRD7-mediated radiosensitivity in NPC cells. The efficiency of BRD7 and METTL3 expression was confirmed by western blotting (Figure 6A), and the CCK-8 and clonogenic survival assays showed that restoring METTL3 expression at least partially reversed the promotive effect of BRD7 overexpression on the cytotoxic effect of irradiation on NPC cells (Figure 6B-D). Moreover, flow cytometric analysis confirmed that restoring METTL3 expression partially reversed the proapoptotic effect of BRD7 on IR-treated NPC cells (Figure 6E). Furthermore, we further investigated the impacts of the BRD7/METTL3 axis on radioresistant NPC cells. As expected, similar results were observed in CNE2-IRR cells and NPC cells (Figure S5A-D). These results demonstrated that BRD7 increases radiosensitivity in NPC cells by reducing the protein stability of METTL3.

### BRD7 increases radiosensitivity by negatively regulating METTL3 expression *in vivo*

**Figure 7:**
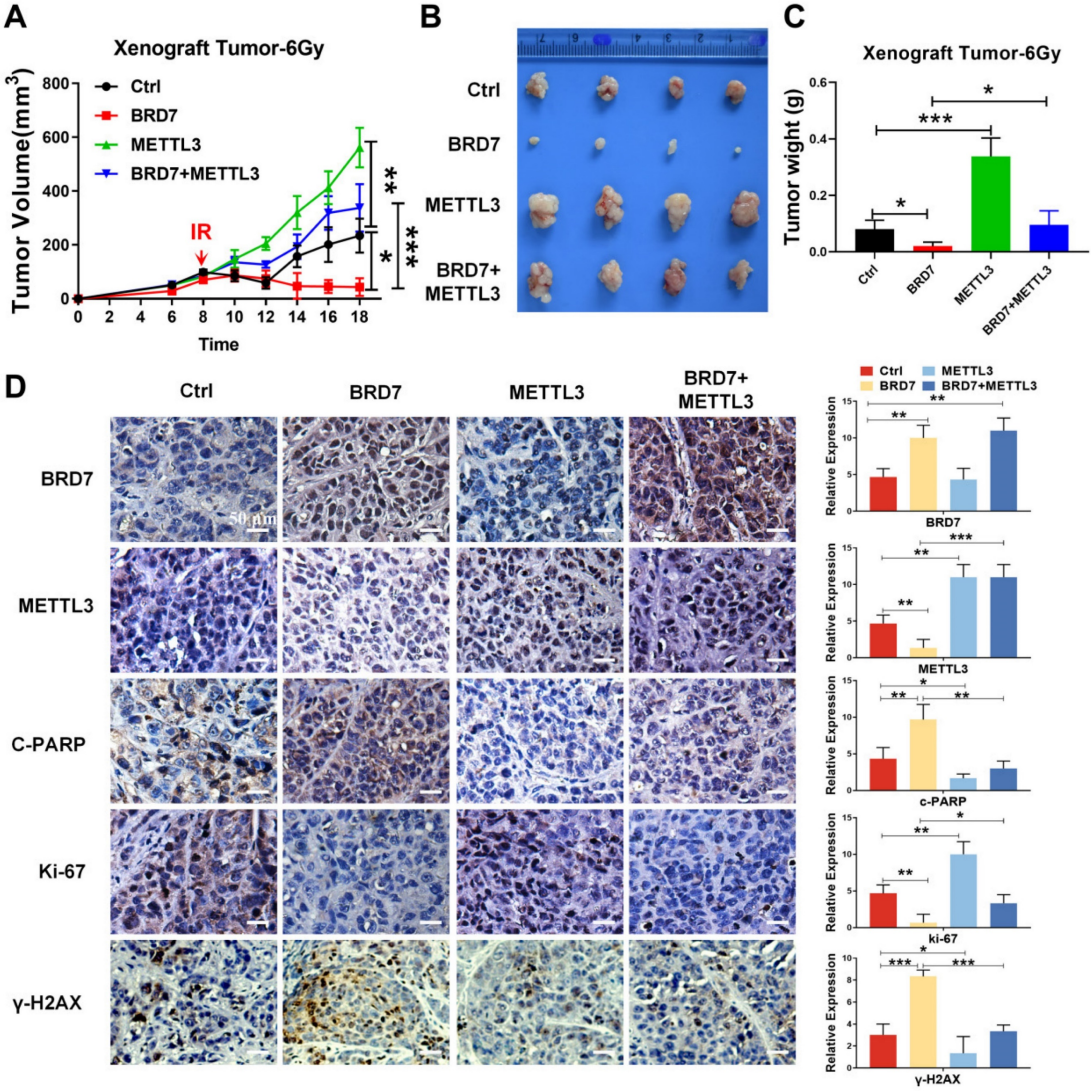
** Restoration of METTL3 expression reverses the radioresistive inhibitory effect of BRD7 of NPC cells *in vivo*.** (A) Growth curve of subcutaneous tumor in tumor-bearing mice. (B) Representative images of collected tumors. (C) Subcutaneous tumor weights in tumor-bearing mice. (D) IHC (DAB staining) for BRD7, METTL3, γ-H2AX, C-PARP, and Ki-67 in tumor tissue sections. Three tumors were analyzed per group. Original magnification, 200×; scale bars represent 50 μm. Error bars represent the mean ± SD. *P<0.05, **P<0.01, ***P<0.001.

To further validate the role of the BRD7/METTL3 axis in the radiosensitivity of NPC *in vivo*, the aforementioned NPC cells were subcutaneously injected into the right flanks of 5-week-old female nude mice for xenograft tumor formation, with a control group, a BRD7 overexpression group, a METTL3 overexpression group, and a BRD7 overexpression followed by METTL3 rescue group established. When the xenograft volume reached ~50 mm^3^, the nude mice were subjected to irradiation (a total of 6 Gy), and the effects were observed on the subsequent days. The tumors began to grow on the 4th-6th days, and the body weight and tumor size were measured once every 2 days. After 18 days, the mice were sacrificed when the tumor volume reached the allowed maximum, and the tumors were stripped and photographed. In line with the results observed *in vitro*, the tumor volume and weight measurements showed that ectopic expression of BRD7 promoted the tumoricidal effect of radiotherapy, i.e., exerted a radiosensitizing effect, while ectopic expression of METTL3 hindered the radiosensitizing effect, and restoring METTL3 expression in BRD7-overexpressing cells antagonized the radiosensitizing effect of BRD7 on NPC cells (Figure 7A-C), and these findings were further confirmed by results of γ-H2AX staining (Figure 7D). In addition, we also detected the expression of proliferation marker Ki67 and the apoptosis marker c-PARP by IHC assay. Taken together, our findings collectively indicated that BRD7 can augment the radiosensitivity of NPC cells by repressing METTL3 expression both *in vitro* and *in vivo*.

### BRD7 is negatively correlated with METTL3 expression and radioresistance in clinical NPC patients

**Figure 8:**
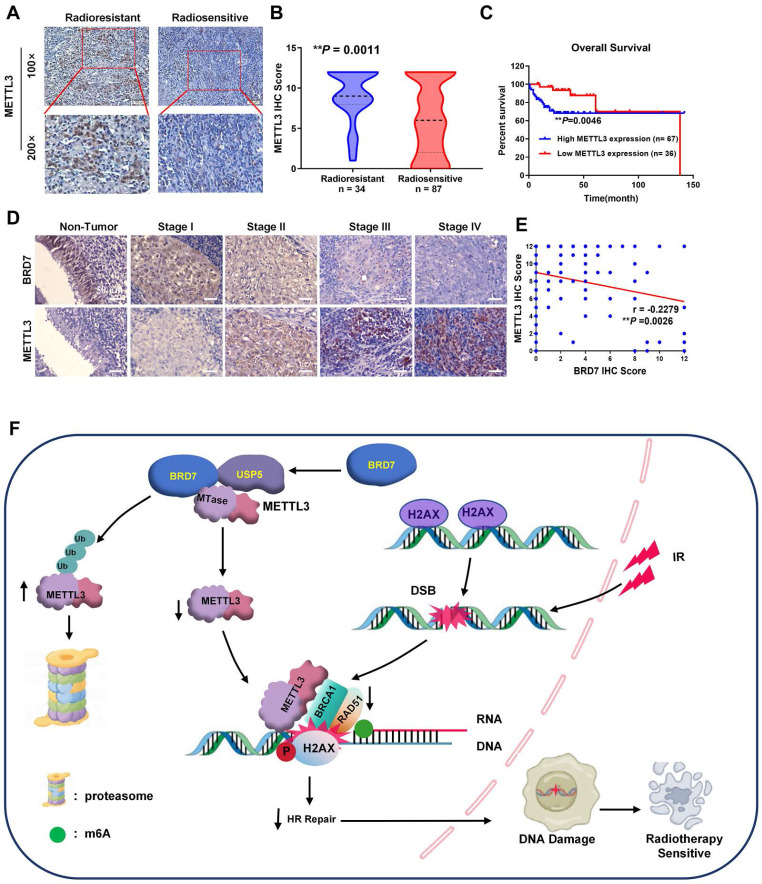
** METTL3 increased is associated with NPC radioresistance and is negatively correlated with BRD7.** (A) and (B) IHC staining of METTL3 protein levels in the radiosensitive and radioresistant specimens of NPC. Original magnification, 100×; scale bars represent 100 μm. (C) Relationship between METTL3 expression levels and prognosis. (D) Representative images of BRD7 and METTL3 IHC staining at different clinical stages (Original magnification, 200×; scale bars represent 50 μm.). (E) Correlation analysis between BRD7 and METTL3 expression was evaluated using Pearson's correlation analysis. (F) A working model based on our results.

Based on the mechanism identified above, we proceeded to measure the METTL3 level in NPC tissues from the abovementioned cohort of patients (Figure 1A). Compared with that in radiosensitive NPC tissues, METTL3 expression was significantly increased in radioresistant NPC tissues, and increased METTL3 expression was positively correlated with NPC radioresistance (Figure 8A-B, Table S2). Survival analyses revealed that a high METTL3 level in NPC tissues correlated with markedly reduced OS in patients (Figure 8C). Furthermore, METTL3 was more highly expressed in NPC tissues than in noncancerous NP tissues, and its expression in tissues of clinical stages III and IV was significantly greater than that in tissues of clinical stages I and II. Conversely, the BRD7 level was lower in NPC tissues than in noncancerous NP tissues, and BRD7 expression was significantly lower in tissues of clinical stages III and IV than in tissues of stages I and II, consistent with the previous results (Tables S1-2, Figures 8D and S6A-C). There was a significant negative correlation between BRD7 and METTL3 expression in these tissues (Figure 8E; Pearson correlation coefficient (r) = -0.2279, *P* = 0.0026). Next, we further analyzed the prognostic value of BRD7 and METTL3 in NPC. Survival analyses demonstrated that low BRD7 expression and high METTL3 expression strongly correlated with reduced OS in patients with NPC (Figure S6C). Collectively, our findings suggest that both BRD7 and METTL3 may play crucial roles in the progression of malignancy and the development of radioresistance in NPC. Targeting the BRD7/METTL3 axis might be a promising therapeutic strategy for clinical radiosensitization of NPC.

## Discussion

We have previously demonstrated that BRD7 is expressed at low levels in both NPC tissues and cells, and it has been proven to be an important tumor suppressor in NPC^9^. In this study, we initially observed significant upregulation of BRD7 expression in biopsies from the radiosensitive group compared to those from the radioresistant group, indicating a potential association between aberrant BRD7 expression and the development of radioresistance in NPC patients. Furthermore, we found that overexpression of BRD7 can enhance the inhibitory effect of radiotherapy on cell proliferation and promote IR-induced apoptosis, thus increasing the radiosensitivity of NPC cells. Radiotherapy is one main treatment modality for solid tumors and exerts effects by killing cancer cells 26. Additionally, it can induce various types of DNA damage, including the most severe type, DNA double-strand breaks. Increased DSB repair proficiency is also a major contributor to radioresistance, and blocking DNA-PK-dependent repair has been shown to increase sensitivity to various anticancer therapeutics 27, 28. Upon DNA damage, H2AX is rapidly phosphorylated at Ser139 (γ-H2AX) in the vicinity of DNA breaks, which triggers the recruitment of DNA repair proteins 29. A linear positive correlation was found between the number of γ-H2AX foci and the number of DSBs; hence, the formation of γ-H2AX foci suggests DSB formation 30, 31. Our results showed that BRD7 increased the formation of γ-H2AX foci and the level of γ-H2AX in NPC cells. Taken together, these findings suggest that BRD7 may promote tumor cell death by inhibiting the repair of radiotherapy-induced DSBs, which may be a potential mechanism through which BRD7 enhances the radiosensitivity of NPC cells.

Previous studies reported that m6A may play an important role in the DNA damage response/repair process 18, 32. Phosphorylated METTL3 can localize to DNA damage sites and modify DNA damage-associated RNAs by m6A methylation, and m6A modifications recruit the m6A reader protein YTHDC1 for protection. The METTL3-m6A-YTHDC1 axis modulates the accumulation of DNA-RNA hybrids at DSB sites. m6A-modified RNAs form DNA-RNA hybrids at DSBs that modulate HR-mediated DSB repair by stimulating the recruitment of repair-related proteins, including BRCA1 and RAD51 19. In our study, we found that overexpression of BRD7 decreased the m6A level and was accompanied by downregulation of the m6A methyltransferase METTL3 in NPC cells. METTL3 is constitutively highly expressed and plays an oncogenic role in a variety of tumors. Moreover, the absence of METTL3 significantly increases the sensitivity of cancer cells and mouse xenograft tumors to DNA-based therapies, such as chemotherapy drugs or radiation 33, 34. In most circumstances, as a core component of the m6A writer complex, METTL3 is vital for m6A modification and has been identified as an oncogene in some hematological diseases and solid tumors 35. In our current study, overexpression of METTL3 significantly decreased the sensitivity of NPC cells to radiotherapy, supporting the hypothesis that METTL3 promotes radioresistance in NPC through modification of HR mediated by m6A, However, the mechanism by which BRD7 is involved in NHEJ repair still requires further investigation.

BRD7 has been shown to be an important transcription factor and is also involved in the posttranslational modification of proteins. For example, BRD7 can negatively regulate the transcriptional activity and expression of components of the c-Myc/miR-141 axis as a coregulator of c-Myc, thereby inhibiting the malignant progression of NPC 9, 36. YB1 has also been identified as an interacting protein of BRD7 in breast cancer; BRD7 binds to the C-terminus of YB1 through its N-terminus, thus promoting YB1 ubiquitination and degradation 37. In our study, we found that BRD7 interacted with the METTL3 protein and reduced its stability via the ubiquitin-proteasome pathway. The dynamic balance between ubiquitination and deubiquitination mediated by E3 ubiquitin ligases and deubiquitinating enzymes controls the ubiquitination, stability and subcellular localization of proteins. USP5 was reported to be a potential deubiquitinating enzyme for METTL3 in melanoma, and both USP5 knockout and the use of the USP5 inhibitor EOAI3402143 (EOAI) reduced METTL3 protein stability in A375 cells 25, 38. In our study, USP5 was able to bind to METTL3, reducing its the ubiquitination and increasing its protein stability, suggesting that USP5 is a potential deubiquitinase for METTL3 in NPC. Moreover, BRD7 can competitively inhibit the binding of USP5 to the METTL3 mTase domain, thereby reducing METTL3 protein stability by increasing its ubiquitination. However, the detailed binding site and whether E3 ubiquitin ligases are involved need to be further investigated.

To further explore whether the radiosensitizing effect of BRD7 in NPC cells depends on the negative regulation of METTL3 protein stability, a series of rescue experiments at the cellular and animal levels were performed. Restoring the expression of METTL3 in BRD7-overexpressing NPC cells at least partially reversed the radiosensitizing effect of BRD7 on NPC cells, suggesting that BRD7 promotes the radiosensitization of NPC cells through negative regulation of METTL3 protein stability. Mechanistically, METTL3 can recruit the HR repair factors RAD51 and BRCA1 to sites of radiotherapy-induced DNA DSB damage through m6A modification to promote HR repair, thereby increasing radioresistance, while BRD7 overexpression inhibits the recruitment of BRCA1 and RAD51 and, in turn, HR repair activity, in NPC cells by decreasing the protein stability of METTL3; this is the mechanism by which BRD7 promotes radiosensitization in NPC cells. However, whether other DNA repair pathways, such as nonhomologous end joining, are involved in the radiosensitizing effect of BRD7 on NPC cells still needs to be further investigated.

## Conclusion

In summary, our current research demonstrated that BRD7 increases radiation-induced DSBs and increases the sensitivity of NPC cells to radiotherapy. Mechanistically, BRD7 decreases METTL3 protein stability by competitively hindering the binding of the deubiquitinating enzyme USP5 to METTL3. Additionally, BRD7 inhibits METTL3-mediated HR repair and radioresistance in NPC cells by prohibiting the recruitment of BRCA1 and RAD51 to sites of IR-induced DNA damage (Figure 8F). Moreover, a positive correlation exists between high BRD7 expression and low METTL3 expression with respect to radiosensitivity and prognosis in NPC patients. Together, our findings reveal a new mechanism of radioresistance in NPC and indicate that targeting the BRD7/METTL3 axis might be a novel therapeutic strategy for NPC radiosensitization.

## Supplementary Material

Supplementary figures and tables.

## Abbreviations

BRD7: bromodomain containing 7
CCK-8: cell Counting Kit-8
Co-IP: co-immunoprecipitation
CQ: chloroquine
DSB: DNA double-strand break
GAPDH: glyceraldehydes phosphate
HR: homologous Recombination
IF: immunofluorescence
IHC: immunohistochemical
IR: ionizing radiation
LC-MS/MS: liquid chromatography tandem MS
M6A: N6-methyladenosine modifications
METTL3: methyltransferase like 3
NHEJ: non-homologous End Joining
NPC: nasopharyngeal carcinoma
UB: ubiquitin
USP5: ubiquitin specific peptidase 5
