# Supplementary material for: BRD7 enhances the radiosensitivity of nasopharyngeal carcinoma cells by negatively regulating USP5/METTL3 axis-mediated homologous recombination repair
Source: Int J Biol Sci. 2024 Nov 11;20(15):6130–45. doi: 10.7150/ijbs.100833 (PMC11628346; doi:10.7150/ijbs.100833)
Supplement: Supplementary file 1 — Supplementary figures and tables. [file ijbsv20p6130s1.pdf]

Supplementary Data

BRD7 enhances the radiosensitivity of nasopharyngeal carcinoma cells by negatively regulating USP5/METTL3 axis-mediated homologous recombination repair

Mengna Li, Jianxia Wei, Changning Xue, Shipeng Chen, Xiangting Zhou, Lemei Zheng, Yumei Duan, Hongyu Deng, Songqing Fan, Wei Xiong, Faqing Tang, Ming Zhou*

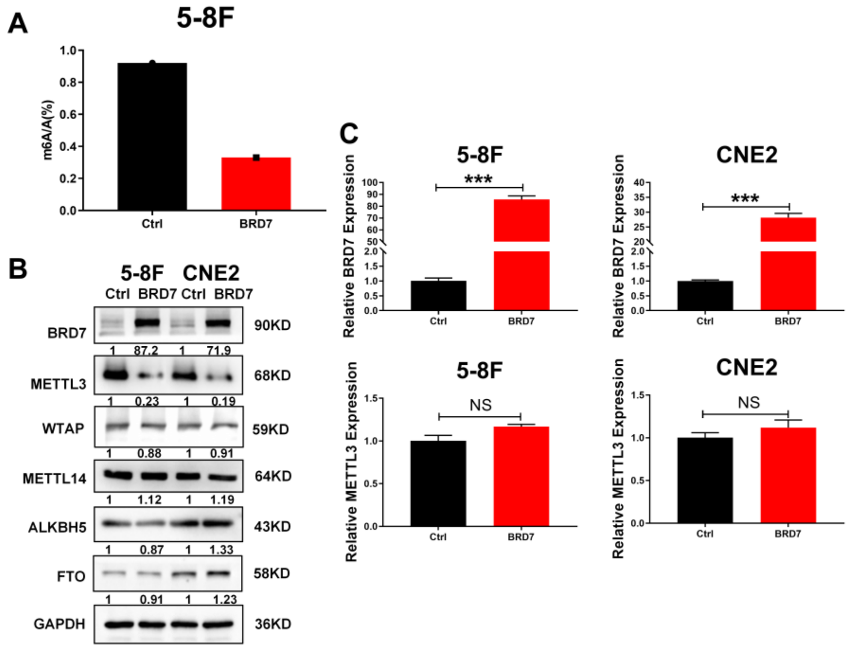

Figure S1. BRD7 inhibits m6A modification in NPC cells by negatively regulating METTL3 protein expression. (A) Liquid chromatography-tandem mass spectrometry (LC-MS) was used to analyze the ratio of m6A/A in NPC cells 5-8F overexpressing BRD7. (B) The expression level of the indicated proteins was determined by western blotting, GAPDH was used as an internal control. (C) The effect of overexpression of BRD7 on METTL3 mRNA level was detected by qPCR, and GAPDH was used as the internal control. Error bars represent the mean ± SD. ****P*<0.001. NS, no significance.

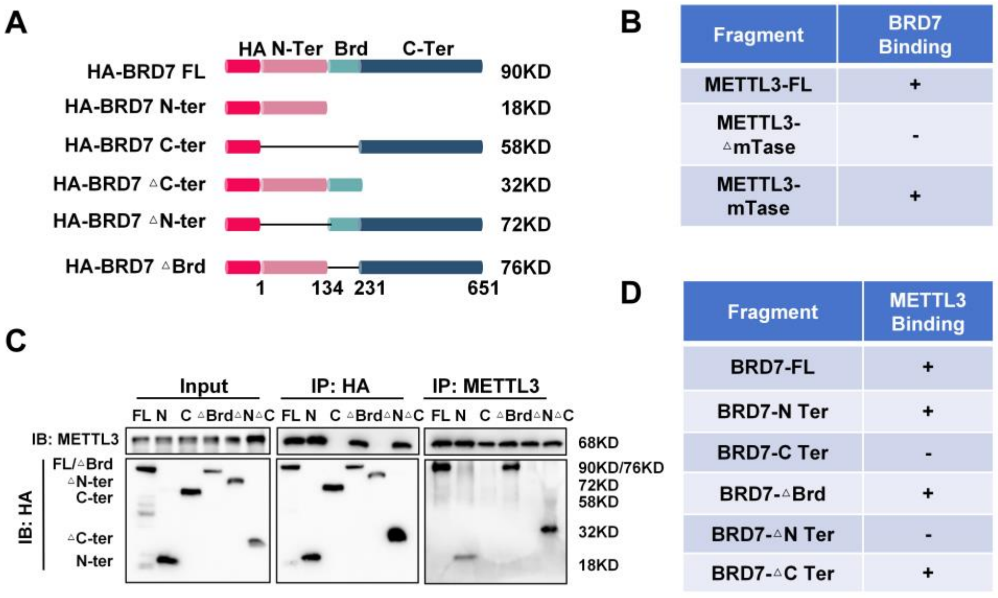

Figure S2. METTL3 directly binds with the N-terminal region of BRD7. (A) Schematic diagram of full-length BRD7 and its truncated derivatives. (B) Qualitative results of the IP assay, where the "+" indicates that the METTL3 truncate interacts with BRD7. (C) Co-immunoprecipitation analysis of Flag-METTL3 with HA-BRD7 truncated mutants. (D) Qualitative results of the IP assay, where the "+" indicates that the BRD7 truncate interacts with METTL3.

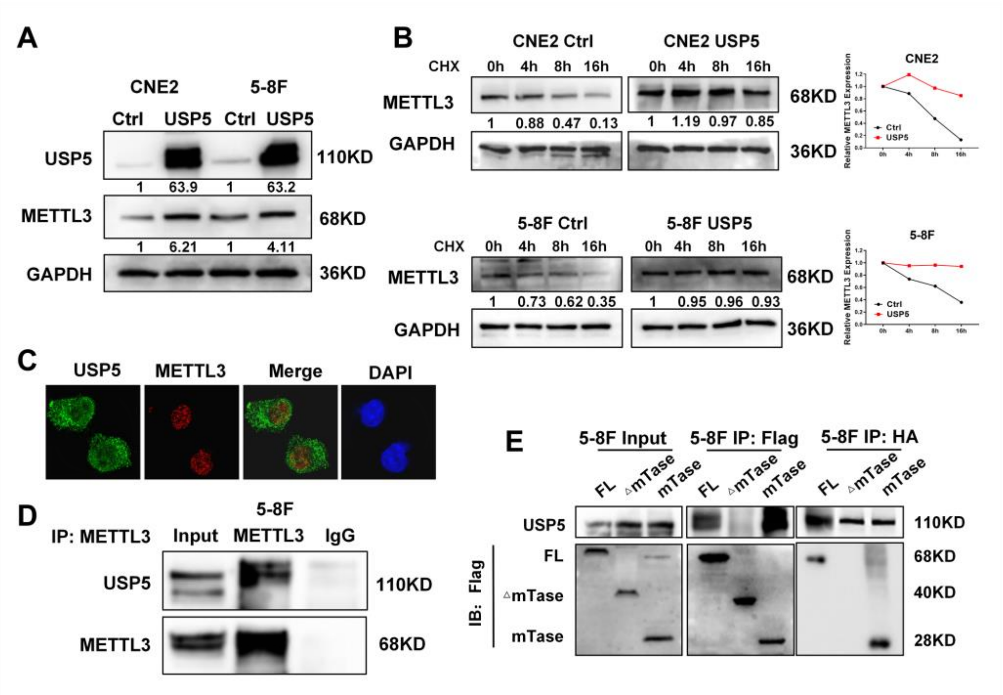

Figure S3. USP5 stabilizes and deubiquitinates METTL3. (A) anti-USP5 and anti-METTL3 antibodies were used to detect the effects of USP5 on the expression of METTL3 protein by western blotting assay, GAPDH was used as an internal control. (B) Western blot analysis of cycloheximide chase to study the effects of USP5 expression on METTL3 protein stability. GAPDH was used as an internal control. (C) Immunofluorescence (IF) analyses of subcellular colocalization of USP5 with METTL3. (D) Co-immunoprecipitation (Co-IP) experiment determined the protein–protein interactions between USP5 with METTL3. (E) Co-immunoprecipitation analysis of HA-USP5 with Flag-METTL3 truncated mutants in 5-8F cells.

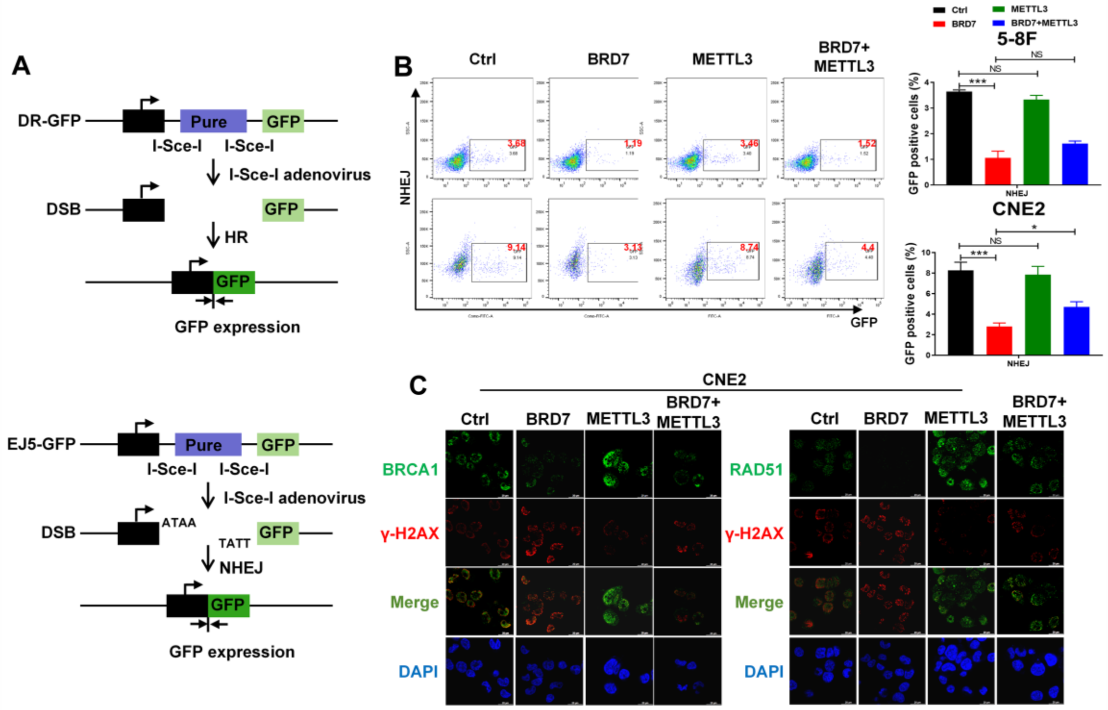

Figure S4. BRD7 inhibition of METTL3-dependent recruitment of BRCA1 and RAD51 to DSBs. (A) Schematic representation of homologous recombination repair and Non-homologous end joining reporting system. (B) Effect of BRD7/METTL3 on NHEJ repair frequency in the NPC cells. GFP-positive cells were quantified 48 hours later by FACS. Error bars represent the mean ± SD. ****P* < 0.001, **P* < 0.05, NS, no significant. (C) Immunofluorescence (IF) to examine recruitment of repair factors BRCA1 or RAD51 to DSB stripe (marked by γ-H2AX).

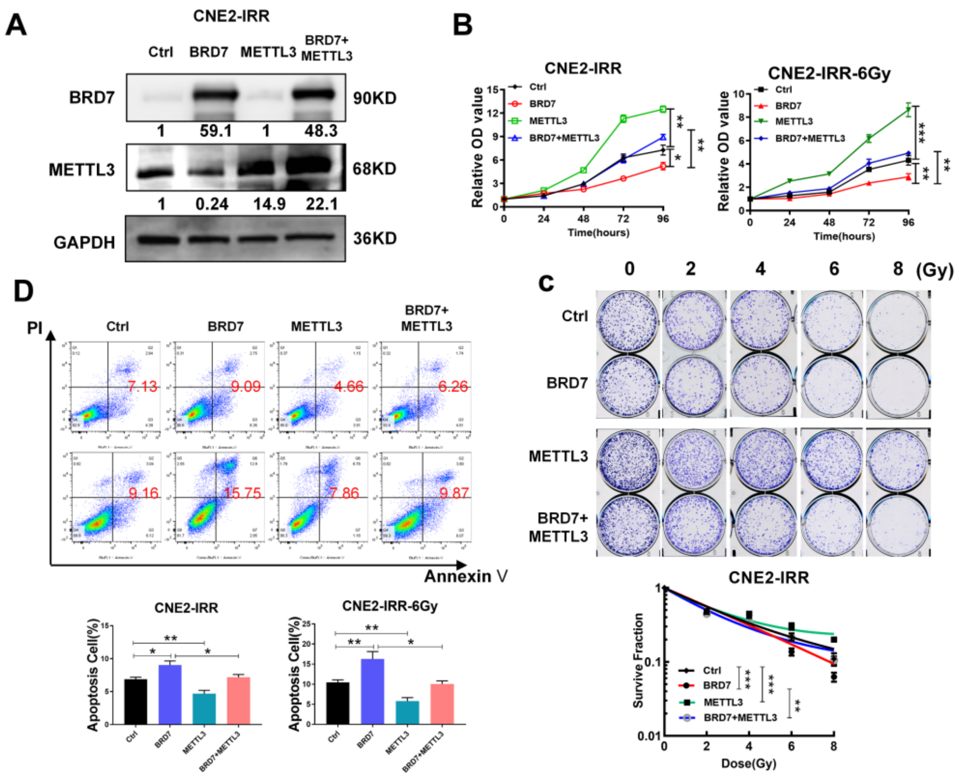

Figure S5. Restoring the expression of METTL3 can partially reverse the proliferation inhibition effect of BRD7 on radiotherapy-resistant NPC cells. (A) Western blotting assay was conducted to detect the expressions of BRD7 and METTL3 in CNE2-IRR, GAPDH was the internal control. (B) The cell viability of BRD7 overexpression, METTL3 overexpression and restoration of METTL3 expression CNE2-IRR cells after radiotherapy at 6 Gy by CCK-8 assays. (C) Survival rate of cells after radiation using clonogenic survival assay. (D) Cell apoptosis analysis via flow cytometry was performed using flow cytometry, cells were treated as described before. Error bars represent the mean ± SD. **P*<0.05, ***P*<0.01, ****P*<0.001. All experiments were performed in triplicate.

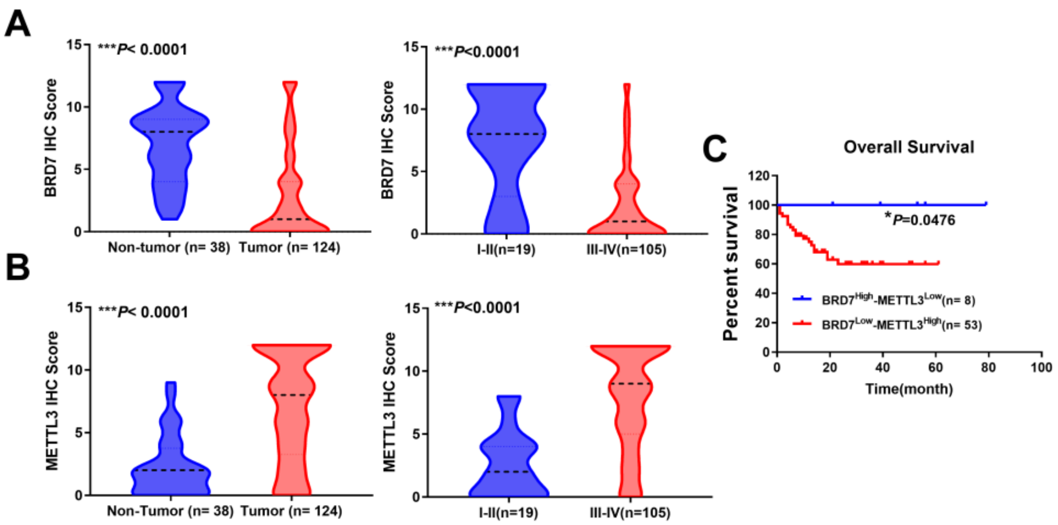

Figure S6. Expression of BRD7 and METTL3 in NPC tissues. (A) and (B) Statistical diagram of BRD7 and METTL3 expression in noncancerous nasopharyngeal tissues and different clinical TNM stages of NPC. (C) Survival based on high BRD7 expression/low METTL3 expression and low BRD7 expression/high METTL3 expression. Error bars represent the mean ± SD. **P*<0.05, ****P*<0.001.

Table S1. Correlation Between BRD7 Expression and Clinical Pathological Features of NPC (n =124).

Variables Features	BRD7 expression		*P*
	Low (n= 96)	High (n= 28)	
Sex			
Male (n= 88)	71 (80.7%)	17 (19.3%)	0.1743
Famale (n= 36)	25 (69.4%)	11 (30.6%)	
Age			
≤41 (n= 24)	14 (58.3%)	10 (41.7%)	0.0128
＞41 (n= 100)	82 (82.0%)	18 (18.0%)	
Radiotherapy			
Radiosensitive (n= 87)	60 (69.0%)	27 (21.0%)	0.001**
Radioresistant (n= 34)	33 (97.1%)	1 (2.9%)	
TNM			
I-II (n= 19)	5 (26.3%)	14 (73.7%)	0.0001***
III-IV (n= 105)	91 (86.7%)	14 (13.3%)	

The following abbreviations were used: High, high expression; Low, low expression. Statistical analysis was performed using the Chi-squared test. *P＜0.05; **P＜0.01.

Table S2. Correlation Between METTL3 Expression and Clinical Pathological Features of NPC (n =124).

Variables Features	METTL3 expression		*P*
	Low (n=43)	High (n=81)	
Sex			
Male (n= 88)	29 (33.0 %)	59 (67.0 %)	0.5285
Famale (n= 36)	14 (38.9 %)	22 (61.1 %)	
Age			
≤41 (n= 24)	9 (30.8 %)	15 (69.2 %)	0.6513
＞41 (n= 100)	34 (34.0 %)	66 (66.0 %)	
Radiotherapy			
Radiosensitive (n= 87)	38 (43.7 %)	49 (56.3 %)	0.0028**
Radioresistant (n= 34)	5 (14.7 %)	29 (85.3 %)	
TNM			
I-II (n= 19)	16 (84.2 %)	3 (15.8 %)	0.0001***
III-IV (n= 105)	27 (25.7 %)	78 (74.3 %)	

The following abbreviations were used: High, high expression; Low, low expression. Statistical analysis was performed using the Chi-squared test. *$P<0.05$; **$P<0.01$; ***$P<0.001$.

Table S3. Correlation Between BRD7, METTL3 Expression and Clinical Pathological Features of NPC (n =124).

Variables Features	BRD7/METTL3 expression		*P*
	Low/High (n= 67)	High/Low (n= 14)	
Sex			
Male (n= 88)	51 (58.0%)	10 (11.4%)	0.7112
Famale (n= 36)	16 (44.4%)	4 (11.1%)	
Age			
≤41（n= 24)	12 (50.0%)	5 (20.8%)	0.1368
＞41（n= 100）	55 (55.0%)	9 (9.0%)	
Radiotherapy			
Radiosensitive （n= 87）	33 (37.9%)	13 (14.9%)	0.0141*
Radioresistant （n= 34）	24 (70.6%)	1 (2.9%)	
TNM			
I-II (n= 19)	1 (5.3%)	12 (63.2%)	0.0001***
III-IV (n= 105)	66 (62.9%)	2 (1.9%)	

The following abbreviations were used: High, high expression; Low, low expression. Statistical analysis was performed using the Chi-squared test. *P<0.05; ***P<0.001.
